# Feasibility and Preliminary Efficacy of a Novel RDoC-Based Treatment Program for Adolescent Depression: “Training for Awareness Resilience and Action” (TARA)—A Pilot Study

**DOI:** 10.3389/fpsyt.2016.00208

**Published:** 2017-01-16

**Authors:** Eva Henje Blom, Olga Tymofiyeva, Margaret A. Chesney, Tiffany C. Ho, Patricia Moran, Colm G. Connolly, Larissa G. Duncan, Lisa Baldini, Helen Y. Weng, Michael Acree, Veronica Goldman, Frederick M. Hecht, Tony T. Yang

**Affiliations:** ^1^Department of Clinical Neuroscience, Karolinska Institutet, Stockholm, Sweden; ^2^Department of Clinical Sciences, Umeå Universitet, Umeå, Sweden; ^3^Department of Psychiatry, Division of Child and Adolescent Psychiatry, UCSF Weill Institute for Neurosciences, University of California San Francisco (UCSF), San Francisco, CA, USA; ^4^Department of Radiology, University of California San Francisco (UCSF), San Francisco, CA, USA; ^5^The Osher Center for Integrative Medicine, University of California San Francisco (UCSF), San Francisco, CA, USA; ^6^Neurosciences Program, Department of Psychology, Stanford University, Stanford, CA, USA; ^7^Department of Human Development and Family Studies, Center for Healthy Minds, University of Wisconsin-Madison, Madison, WI, USA; ^8^Department of Family Medicine and Community Health, Center for Healthy Minds, University of Wisconsin-Madison, Madison, WI, USA

**Keywords:** adolescent depression, Research Domain Criteria (RDoC), non-pharmacological, novel treatment development, emotion regulation, autonomic regulation, mindfulness-based, yoga-based

## Abstract

**Background:**

The novel group treatment program Training for Awareness, Resilience, and Action (TARA) was developed to target specific mechanisms based on neuroscience findings in adolescent depression and framed within the National Institute of Mental Health Research Domain Criteria. TARA contains training of autonomic and emotional self-regulation, interoceptive awareness, relational skills, and value-based committed action.

**Methods:**

We performed a single-arm trial to test the feasibility and preliminary efficacy of TARA in reducing depression and anxiety levels and assessed whether the specific targeted domains of function reflected the hypothesized symptom change. Twenty-six adolescents (14–18 years old, 7 males and 19 females) participated in the 12-week group program. Assessment was performed before (T0), immediately after (T1), and 3 months after the end of TARA (T2).

**Results:**

Significant improvement was seen in depression symptoms (Reynolds Adolescent Depression Scale Second Edition) between T0–T1 (*t*-value = −3.56, *p* = 0.002, CI = −6.64, −1.77) and T0–T2 (*t*-value = −4.17, *p* < 0.001, CI = −11.20, −3.75) and anxiety symptoms (Multidimensional Anxiety Scale for Children) between T0–T1 (*t*-value = −2.26, *p* = 0.033, CI = −4.61, −0.21) and T0–T2 (*t*-value = −3.06, *p* = 0.006, 95% confidence interval = −9.02, −1.73). Significant improvements in psychological flexibility, sleep, and mindfulness skills were also found between T0 and T2.

**Limitations:**

The sample size was small without a control condition. The pilot design did not allow for testing the hypothesized brain changes and effect of TARA on relevant systemic biomarkers.

**Conclusion:**

TARA is feasible in a sample of clinically depressed and/or anxious adolescents and preliminary efficacy was demonstrated by reduced depression and anxiety symptoms. The specific symptom and behavioral outcomes corresponded well with the hypothesized mechanisms of change.

## Introduction

More effective treatment of adolescent depression is critically needed given that global projections by the World Health Organization identify major depressive disorder (MDD) as the leading cause of disease burden by 2030 (1). This is partly explained by a combination of the age distribution of the global population and the sharp increase of onset of MDD during the teenage years, with a current lifetime prevalence of 11.0% in this age group (2). Early onset depression predicts a fourfold increase in the risk of developing adult depression and is associated with higher risk of future negative health outcomes and suicide (3). Furthermore, the relapse rate 5 years after remission is 70% (4).

One way to address this problem is to close the current gap in the translation between the rapidly growing body of literature on neural mechanisms characterizing adolescent depression and the development of targeted treatments. Such an attempt has been made through the development of a novel treatment approach for adolescent depression *Training for Awareness, Resilience, and Action* (TARA) (5) aligned with the Research Domain Criteria (RDoC) of the National Institute of Mental Health (NIMH) (6). The preliminary conceptual framework of TARA has been previously described (5). Our approach to use the RDoC in combination with developmental aspects of the brain as the foundation for creating a treatment model for adolescent depression is pioneering work. We appreciate the complexity of the different neurocircuits implied in adolescent depression and the difficulty to disentangle them, since overlaps between different domains and constructs in the RDoC matrix are common. We have therefore reduced our targets for the TARA treatment model to specific neural mechanisms rather than all of the neurocircuitry suggested in each construct. We were inspired by a similar development of an RDoC-based approach for treatment of geriatric depression that was published in the journal Molecular Psychiatry (7). In creating the TARA intervention, we started by identifying the major RDoC domains of function involved in adolescent depression and then organized them in a way that gave priority to domains thought to be driving the psychopathology. The next step was to enhance the translation into an effective clinical intervention for adolescent depression. Thus, we integrated approaches from several different paradigms and traditions based on their feasibility, efficacy and congruence with our neuroscience-based scientific theory of change.

Importantly, the DSM diagnostic criteria for adolescent MDD have low diagnostic validity and specificity, resulting in unclear diagnostic boundaries especially with regard to generalized and social anxiety (8–13). As a result, symptoms of generalized and social anxiety are highly comorbid with depression symptomatology in this age group (14–17). This has been specifically addressed in the DSM5, which states “*in terms of genetic risk, family, twin, and high-risk transmission studies all indicate that Generalized Anxiety Disorder (GAD) and MDD share some, if not most, of their genetic risk factors*” and a new diagnostic entity of mixed depression and anxiety has been introduced. In the RDoC matrix, it is also evident that generalized and social anxiety and depression symptomatology are linked to dysfunction in the same neurocircuitry, such as sustained limbic hyperreactivity (18–20) and reduced adaptive flexibility of the default mode network (DMN) (20–22). Consequently, we chose to include participants with symptoms of depression and/or generalized and social anxiety in this study and hypothesized that the TARA intervention should impact both depressive symptomatology and symptoms of generalized and social anxiety.

In this pilot study, we aimed to test feasibility, acceptability to participants, and clinical plausibility of the TARA intervention before moving on to more intensive neural assessment. As a clinically relevant proxy for the hypothesized brain changes that we outline below, we used self-reported psychiatric symptom and behavioral changes in alignment with the RDoC criteria.

Previous and current fMRI studies from our group and others consistently show that depressed adolescents demonstrate increased hyperactivation of the amygdala, a key limbic structure involved in emotion generative processing (18) and aberrant functional connectivity between the amygdala and other emotional regulation areas (19, 23, 24). This may be related to sustained increase of stress levels with autonomic dysregulation, including a sympathetic overdrive and a decreased parasympathetic tone (25, 26). This suggests that vagal afference could be a therapeutic possibility (27, 28). We hypothesized that targeting the limbic system, by practicing to increase the vagal afference by a “bottom-up” approach, could be an effective method to treat depression. Maybe specifically so in adolescence, when the dorsolateral prefrontal cortex is not yet developed to full adult capacity and “top-down” cognitive control is more challenging.

Consequently, *the first target* of the TARA treatment is to increase vagal afference. Based on the literature of respiratory biofeedback (29–31), autonomic regulation (26), yoga-based interventions (32), and vagus nerve stimulation (28), we aimed to promote vagal and sensory afference through breathing practices and slow synchronized movement during the first module (weeks 1–3) (5). We hypothesized that this would correspond to decreased feelings of stress and physical symptoms of depression and anxiety (33) in addition to improved emotional self-regulatory abilities and sleep.

*The second target* in TARA is practicing the ability to shift neural activation away from the DMN, which has been found to be significantly altered in fMRI studies of depressed adolescents (34). The DMN includes medial cortical structures, such as the ventromedial prefrontal cortex and the posterior cingulate cortex, and it is critical to negative self-referential processing such as rumination and worrying known to go awry in depression (35–38). The ability to notice when rumination or worrying occurs and then consciously switch from this DMN-dominant brain state to one focused on present moment sensory awareness has been suggested to have a therapeutic effect in adults with mood disorders (39). During the second module (weeks 4–6), TARA participants practiced noticing negative self-referential processing and then shifting to present moment sensory and interoceptive awareness (39–41). By adding practices of identifying, labeling, and expressing emotional processes, we hoped to further decrease depressive and anxious symptoms (42, 43). The corresponding hypothesized outcome was decreased rumination and generalized anxiety.

A large body of literature shows that interpersonal stress and social rejection are the strongest proximal risk factors for depression (44). *The third target* was therefore to bring the acquired skill set into managing of emotions during social interactions, which is a relevant real world challenge for many teenagers with depressive problems. Recognizing emotional triggers in oneself and others, empathetic listening, effective communication, and compassionate responses to oneself and others were practiced in the third module (weeks 7–9). The corresponding hypothesized outcome was decreased social anxiety.

Finally, fMRI studies suggest that regulation of mood states depends on interactions, not only as previously described, between prefrontal areas and limbic networks, but also between the prefrontal areas and striatal nodes (45). Depressed adolescents show disrupted balance of cortico-striatal circuit function, with low striatal response and high medial prefrontal response to reward (45). A recent diffusion MRI-based study also revealed a right caudate-centered sub-network of lower structural connectivity in adolescents with MDD (46). Both acute and chronic stress also appears to increase reward deficits, which, in turn, raises the risk for depression (47). Due to these findings, *the fourth target*, addressed in the fourth and last module (weeks 10–12), was to increase behavioral activation guided by intrinsic reward, inspired by techniques in acceptance and commitment therapy (48). Here, TARA participants formulated a personal strategy for how to live a life aligned with their values, while challenging patterns of experiential avoidance, by using practices of top-down cognitive control of affective responses. The hypothesized behavioral outcomes were decreased experiential avoidance, increased committed action, and improved behavioral effectiveness amidst distressing emotional experiences.

In addition to the described neural constructs targeted in the development of TARA, contextual factors hypothesized to contribute to depressive pathophysiology through increased perception of sustained threat were addressed. These included discussions about bullying, climate change, and other factors about which the participants were concerned (49).

The overall aims for this single-arm pilot study were to test the feasibility, acceptability, and preliminary efficacy of the TARA intervention in overall reduction of depression and generalized and social anxiety symptom scores and to assess whether the targeted domains of function were associated with the hypothesized symptom and behavioral changes.

## Materials and Methods

### Intervention Format of TARA

The TARA intervention took place over 12 consecutive weekly sessions, lasting 90 min each, in a group of up to 12 participants. The first session aimed to create a sense of safety by introducing the group members; establishing clear guidelines; investigating attitudes, opinions, and previous experiences of group processes; and introducing contemplative practices. During the sessions, participants sat in a circle on yoga mats with the two facilitators opposite each other. First, facilitators “opened the circle” by ringing a bell and briefly checking-in with everyone. Next, participants were guided through 5 min of gentle breathing practices. Yoga-based movement followed, consisting of a flow of positions synchronized with the breath. Finally, facilitators guided a meditation practice that focused mainly on interoceptive and sensory awareness that ranged from 1 min in the first session to 10–15 min duration toward the end of the 12-week intervention period. After a short, informal break with snacks, there was time for feedback and questions regarding the home practice from the previous week. This was followed by a brief psycho-educational presentation including small and large group exercises and discussions. The sessions concluded with the facilitators describing the home practices for the coming week and “closing of the circle,” when the participants gathered their attention and then had the opportunity to name any reflections from the session.

Development of the TARA program was inspired by the structure and content of mindfulness-based approaches but is fundamentally different in several ways. First, manipulation of the breath and synchronized slow movements were used to improve emotional self-regulatory skills, rather than a primary focus on acceptance of emotional experience through metacognition. There was a focus on “real world” relevance for the adolescents, and trans-generational dialog and inquiry were emphasized. We also aimed to create full transparency of the rationale for each practice, often with a scientific background explained in the form of a slide presentation. Participation and relational practices were emphasized, as well as practices of validating and skillfully expressing emotional content. Value-based committed action was an extended goal of the curriculum, not only equanimity and personal well-being, as is often the case in mindfulness-based interventions. Time was spent contextualizing depressive symptoms and investigating the negative impact that the cultures and systems we live in have on personal health. Home practice of TARA skills was encouraged, with audio tracks of breathing instructions and short, guided meditations provided to participants.

### Facilitator Training and Setup

Each class had two facilitators, together covering expertise in contemplative practices and clinical child psychology or psychiatry. The first author, who developed the TARA intervention, observed the classes to provide ongoing supervision and training to the teachers and monitor fidelity of implementation of the TARA protocol, both in terms of content adherence and fidelity to the process of delivery of the different modalities of the intervention. The emphasis was not only in teaching specific content but also in modeling a collaborative, inclusive, non-judgmental, and supportive attitude.

### Participants and Recruitment

Participants were recruited through the San Francisco United School District’s public high school Wellness Centers, community sites throughout the San Francisco Bay Area, and through fliers and outreach by study staff. In this pilot study, our team worked to identify the appropriate inclusion criteria for the study, settling on 14- to 18-year-old adolescents still in high school, with clinically significant depressive and/or anxious symptomatology as measured by the Children’s Depression Rating Scale-Revised (CDRS-R > 35) (50, 51) and/or by the Multidimensional Anxiety Scale for Children (MASC *T*-score >56) (52), respectively. All participants were required to be under the care of a physician or mental health provider. Exclusion criteria were mainly conditions that would prevent effective group participation, i.e., current comorbidity of psychosis, severe anorexia nervosa, acute posttraumatic stress disorder, severe self-mutilation, severe suicidal ideation, or any suicide attempt in the past 3 months, lifetime comorbidity of bipolar disorder, low-functioning autism spectrum disorder, intellectual disability (estimated IQ < 80), and substance dependence. We excluded non-English speakers because the TARA groups were conducted in English. Individuals with ongoing practice of meditation and/or yoga of >20 min two times per week or more for the past 2 months were also excluded. One participant, with no suicidal ideation at baseline, was permitted to enroll in the study despite being treated concurrently with Dialectical Behavior Therapy.

All participants provided informed written assent and their parent(s)/legal guardian(s) provided informed written consent, in accordance with the Declaration of Helsinki. The Human Research Protection Program of the University of California San Francisco approved all study procedures. Potential participants and their parents/guardians (if <18 years old) first completed a semi-scripted phone screening, designed to elicit preliminary inclusion/exclusion information and to provide additional information about TARA. The study procedures were described to ensure the candidates understood the commitment involved. If they were eligible and still interested, they were scheduled for the first intake interview. An interviewer-administered depression and suicide assessment was then performed, and self-report scales for depression and anxiety symptoms, and demographics were completed. A diagnostic assessment was administered online to rule out comorbidities that would exclude participation.

Immediately prior to the start of the intervention (T0), a baseline visit was scheduled for participants to complete self-report questionnaires. The same assessment was repeated immediately after the TARA intervention (T1, 3 months later) and at 6-month follow-up from baseline (T2). Participants were also asked to complete session-by-session ratings and invited to participate in a post TARA focus group to discuss their experience and receptivity to the TARA intervention with an external interviewer and offer suggestions for improvement. The focus group session was audio-recorded and then transcribed.

### Measures

#### Primary and Secondary Outcome Measures, Self-Report Scales

Reynolds Adolescent Depression Scale Second Edition (RADS-2) was the primary depression outcome measure. The RADS-2 has excellent psychometric properties and is validated in depressed adolescents (53). It contains 30 items, providing 4 subscales measuring different dimensions of depression: Dysphoric Mood, Anhedonia/Negative Affect, Negative Self-Evaluation, and Somatic Complaints. Raw scores range from 30 to 120.

Multidimensional Anxiety Scale for Children (MASC) was the primary outcome anxiety measure and was also used to access eligibility as it is considered the best normed and psychometrically strong self-report anxiety scale for use with adolescents (52). The MASC contains 39 items with a total raw score ranging from 0 to 117. It assesses four dimensions of anxiety: physical symptoms, harm/avoidance, social anxiety, and separation/panic.

Insomnia Severity Index (ISI) was a secondary measure and is a brief 7-item assessment scale for insomnia (54) with scores ranging from 0 to 28.

Child and Adolescent Mindfulness Measure (CAMM) was also a secondary measure and is a 10-item self-assessment scale based on a Likert scale with possible scores ranging from 1 to 100, which is shown to have adequate internal consistency and be a useful measure of mindfulness skills for school-aged children and adolescents (55).

Avoidance and Fusion Questionnaire for Youth (AFQ-Y) was the final secondary measure and is an 8-item measure of psychological inflexibility fostered by (a) cognitive fusion, (b) experiential avoidance, and (c) inaction or behavioral ineffectiveness in the presence of unwanted internal experiences (56). Measured on a 5-point Likert Scale with possible scores ranging from 0 to 32.

#### Secondary Outcome Measures, Clinician Assessment

Children’s Depression Rating Scale-Revised (CDRS-R) was used as a secondary depression outcome and was also used to assess study eligibility. It is a standardized rating scale based on a semi-structured interview and provides an efficient way to diagnose childhood depression and monitor treatment response (50). Specifically trained staff performed this assessment, and it was videotaped for quality control by a child psychiatrist.

#### Baseline Assessments

Development and Well-Being Assessment (DAWBA) is an Internet-based semi-structured diagnostic interview compatible with the psychiatric diagnostic criteria of the DSM-IV for 5- to 17-year-old individuals. DAWBA has consistently generated sensible estimates of prevalence and association with risk factors (57). When compared to clinical diagnoses, DAWBA diagnoses have good validity (58). DAWBA is used in large multicenter neuroimaging studies in Europe, such as the Imagen study (59).

Columbia Suicidality Severity Rating Scale (CSSRS) was used to screen for suicidality. This widely used measure assesses the full range of evidence-based suicidal ideation and behavior items (60).

Hollingshead Four Factor Index of Socioeconomic Status (61) was used to gather data on race, parents’ marital status, employment status, educational attainment, and occupation.

Childhood Trauma Questionnaire (CTQ) (62) is a 28-item self-report scale used to measure history of child abuse and neglect. CTQ has dichotomous clinical cutoff scores for the five subscales that differentiate between presence or absence of significant physical abuse, physical neglect, sexual abuse, emotional abuse, and emotional neglect (63). Reliability and validity are well established, and the total CTQ scale is considered capable of adequately capturing a broad dimension of childhood maltreatment (64).

#### Monitoring Instruments

The Child Outcome Rating Scale (CORS) and Child Session Rating Scale (CSRS) are 4-item self-assessments using a 10-cm visual analog scale (65), with higher scores indicating better functioning/experience. Participants completed the CORS before each session, rating how they had been doing individually, in the family, in school, and overall, over the prior week. The CSRS, a measure of working alliance, was completed after each session. Participants rated the session in terms of how much they felt listened to, how important the content and activities were to them, how much they liked the session, and their overall experience.

### Statistical Methods

Means and SDs were calculated for each self-report and clinician rating at each time point and calculations were based on intended to treat analyses. To assess change in the clinical and self-assessment scores from T0 to T1 and from T0 to T2, we used hierarchical models of change, including all three time-points as a linear trend. Effect sizes (ES) were calculated based on proportion of variance between T0 and T1 and between T0 and T2, accounted for by the time effect. Dropouts were defined as clinical trial dropouts, i.e., having missed more than 50% of the classes. Baseline scores of RADS-2 and MASC were used in logistic regression to predict clinical dropout. SAS 9.4 was used for all analyses.

## Results

### Attendance and Retention

**Figure 1:**
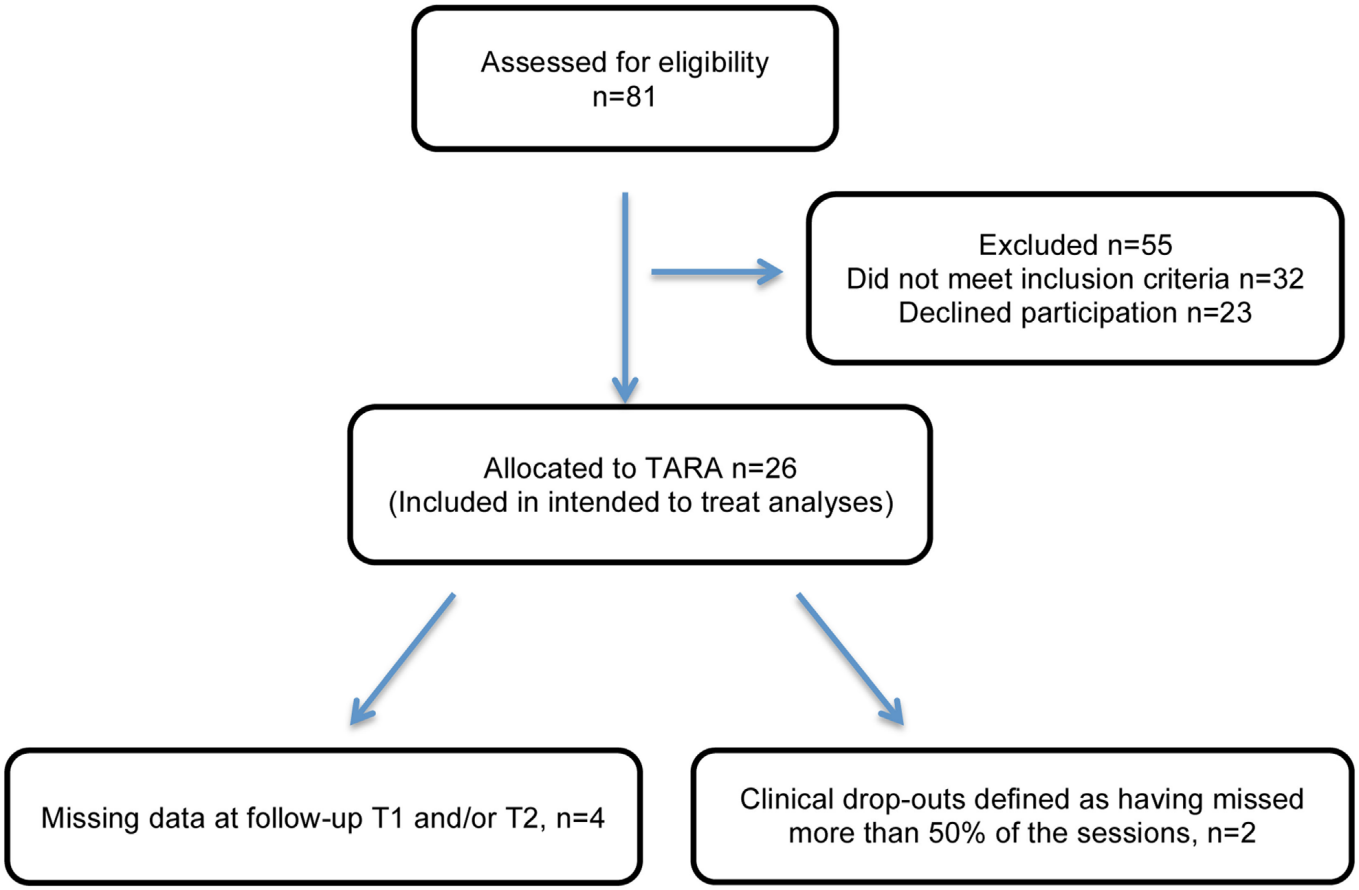
**Consort flow diagram showing the recruitment process**.

Twenty-six participants (mean age 15.6, range: 14–18 years, 7 boys and 19 girls) were eligible and enrolled in the TARA program in three consecutive groups: the first (Group A, *n* = 4) started in May 2014, the second (Group B, *n* = 11) in January 2015, and the third (Group C, *n* = 11) in April 2015. Groups A and B had the same teachers and Group C had a new set of teachers. Two of the participants (8%) missed more than 50% of the sessions and the mean attendance rate per session was 78.5% of the participants (Figures 1, S1). The mean proportion of sessions attended by individual participant was on average 9.5 of the 12 sessions, i.e. 79.2% of the total sessions (Figures S1, S2).

### Sample Baseline Characteristics

#### Race and Socioeconomic Status

Our sample contained participants with the following racial distribution: White 46%, Hispanic/Latino 15%, Asian 12%, Native Hawaiian 4%, African-American 0%, and multiracial 23% (Table 1). Parents’ level of education was diverse and ranged from one or both parents with less than a high school education (*n* = 6) to both parents completing graduate or professional training (*n* = 13). The majority of participants (*n* = 23, 88%) had at least one employed parent.

**Table 1:** **Baseline sample characteristics of the three Groups A–C of ***Training for Awareness, Resilience, and Action*** (TARA), including mean total ***T*** scores and SD of self-assessment with Reynolds Adolescent Depression Scale Second Edition (RADS-2) and Multidimensional Anxiety Scale for Children (MASC), mean scores and SD of Avoidance and Fusion Questionnaire for Youth (AFQ-Y), Insomnia Severity Index (ISI), the Child and Adolescent Mindfulness Measure (CAMM), interview assessment with Children’s Depression Rating Scale-Revised (CDRS-R), and finally socio-demographic data gathered by Hollingshead Four Factor Index of Socioeconomic Status**.

Sample characteristics	Group A *n* = 4	Group B *n* = 11	Group C *n* = 11

	Mean score (SD)	Mean score (SD)	Mean score (SD)
RADS-2 total *T* score	68.0 (8.68)	65.4 (11.07)	57.3 (9.17)
MASC total *T* score	59.5 (2.65)	71.9 (9.16)	59.5 (9.33)
ISI	–	14.8 (5.83)	6.8 (4.94)
CAMM	28.8 (3.87)	24.4 (6.09)	30.7 (8.79)
AFQ-Y	48.0 (19.6)	53.1 (15.3)	40.7 (13.6)
CDRS-R	70.5 (10.27)	68.0 (8.54)	63.8 (2.82)
Ratio boys/girls	2/2	1/10	4/7
Both parents born in US	2/4	8/11	8/11
Participant born in US	4/4	10/11	11/11
Race: White/Hispanic/Asian/African-American/Multiracial	2/0/1/0/1	3/4/2/0/2	7/0/0/0/3 + 1 Native Hawaiian
Lives with biological father and/or mother	3/4	10/11	9/11
Parents’ divorced	2/4	3/11	5/11
Parents’ educational level was post high school	0/4	5/11	7/11
Mean household income, USD/year (range)	35,000 (<10,000–80,000)	125,000 (<10,000–500,000)	96,000 (35,000–450,000)

#### Lifetime Trauma Exposure

The baseline CTQ total scores ranged from 36.25 to 96.82, with a mean value of 55.79 and SD of 14.39 indicating presence of significant lifetime abuse and neglect based on the CTQ cutoffs.

#### Suicidality

During the CSSRS, four participants reported a lifetime history of suicide attempts, two of whom had been psychiatrically hospitalized. Fifteen participants reported a prior history of suicidal ideation, and 10 had self-harming behavior. In the 2 weeks prior to the baseline interview, seven participants reported experiencing suicidal ideation which was sporadic and not assessed as severe, two participants reported self-harming behavior without suicidal ideation; no one reported recent suicide attempt. These seven participants were not considered fulfilling the exclusion criterion of severe suicidal ideation and therefore enrolled in the study.

#### Comorbid Psychiatric Disorders

Based on prior assessment and validation with DAWBA, 17 of the participants had a DSM-IV diagnosis of MDD, 14 met criteria for a DSM-IV anxiety disorder (AD). Eleven participants had comorbidity of both MDD and an AD, and six participants had neither a DSM-IV diagnosis of MDD or AD but scored above the described cutoff on CDRS-R and/or MASC (Figure S3). In addition to MDD and/or AD, 2 participants had a comorbid eating disorder and 10 had comorbid ADHD.

#### Concurrent Treatment

Nearly all (88%) of the participants were receiving psychotherapy or counseling at the time of TARA enrollment, with duration ranging from 2 to 121 months. Seventeen of the participants were taking psychotropic medication, which had been ongoing for 3–24 months prior to entering the TARA program. Twelve participants (46%) were treated with some kind of antidepressant medication: 10 with selective serotonin reuptake inhibitors (sertraline, fluoxetine, and escitalopram), two with serotonin–noradrenaline reuptake inhibitors (venlafaxine and duloxetine), one with a serotonin receptor antagonist and reuptake inhibitor (trazodone). In addition, 1 participant was treated with a benzodiazepine (clonazepam), 1 with a mood stabilizer (lamotrigine), and 10 with central stimulant drugs (9 with metylphenidate and one with atomoxetin). Sixteen (62%) of the participants had failed to respond to psychological treatment for 6 months or more and 5 of the participants (19% of the 26 participants) had failed to respond to antidepressant medication for 6 months or more.

#### Group Differences at Baseline

Sample characteristics at baseline in Groups A–C see Table 1.

### Primary Outcomes

**Figure 2:**
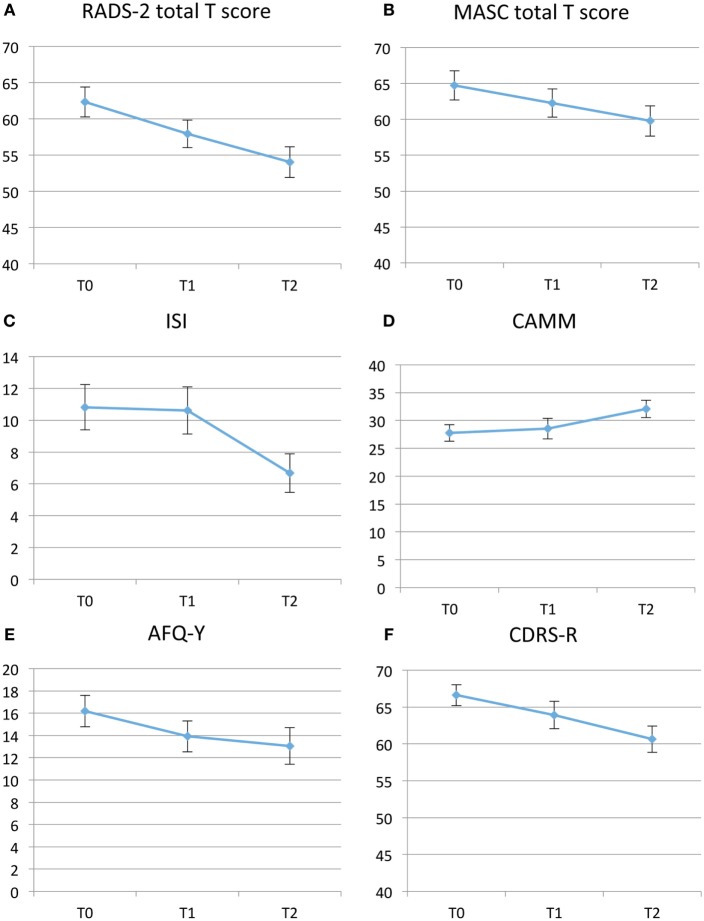
**(A–F)** Self-assessment mean results pre-*Training for Awareness, Resilience, and Action* (TARA) (T0), post-TARA (T1), and at 3-month follow-up after TARA (T2). Note that the *y*-axis in Panel **(A,B,F)** range from 40 to 70. **(A)** Depression symptoms with Reynolds Adolescent Depression Scale Second Edition (RADS-2), total *T* scores. **(B)** Anxiety symptoms with Multidimensional Anxiety Scale for Children (MASC), total *T* scores. **(C)** Insomnia with Insomnia Severity Index (ISI), total scores. **(D)** Emotional self-regulation and “mindfulness skills” with the Child and Adolescent Mindfulness Measure (CAMM), total score. **(E)** Avoidance and Fusion Questionnaire for Youth (AFQ-Y) total scores. **(F)** Clinician assessed Children’s Depression Rating Scale-Revised (CDRS-R).

Depression symptom severity as measured by the RADS-2 total *T* scores showed significant improvement from T0 to T1 with a mean difference = −4.21, *t*-value = −3.56, *p* = 0.002, ES = 0.53, 95% confidence interval (95% CI): −6.64 to −1.77 and T0–T2 with a mean difference = −7.48, *t*-value = −4.17, *p* < 0.001, ES = 0.83, 95% CI: −11.20 to −3.75. Anxiety symptoms as measured by MASC between T0 and T1 showed mean difference = −2.41, *t*-value = −2.26, *p* = 0.033, ES = 0.20, 95% CI: −4.61 to −0.21 and between T0 and T2 mean difference = −5.37, *t*-value = −3.06, *p* = 0.006, 95% ES = 0.38 CI: −9.02 to 1.73 (Table 2; Figures 2A,B). The RADS-2 subscale Negative Self-Evaluation showed the biggest ES of 0.87 between T0 and T2. Total *T* score of RADS-2 and MASC at baseline did not predict dropout during the TARA intervention.

**Table 2:** **Difference in mean scores, t-values, p-values, and 95% confidence interval (95% CI) between pre-***Training for Awareness, Resilience, and Action*** (TARA) (T0) and post-TARA (T1) and between T0 and 3 months after the end of TARA (T2)**.

	T0 baseline–T1 post-TARA (immediate post-intervention; 3 months after study entry)				T0 baseline–T2 post-TARA (3-month post-intervention follow-up; 6 months after study entry)			
	
Variable	Difference	*t* (*df* = 24)	*p*	95% CI	Difference	*t* (*df* = 21)	*p*	95% CI
RADS-2 total	−4.21	−3.56	0.002	(−6.64, −1.77)	−7.48	−4.17	<0.001	(−11.20, −3.75)
RADS-2 anhedonia/negative affect	−4.50	−2.54	0.018	(−8.15, −0.85)	−5.99	−2.37	0.027	(−11.23, −0.74)
RADS-2 dysphoric mood	−2.17	−1.45	0.160	(−5.26, 0.92)	−5.81	−2.84	<0.010	(−10.06, −1.56)
RADS-2 negative self-evaluation	−4.85	−3.81	<0.001	(−7.47, −2.22)	−7.28	−4.17	<0.001	(−10.92, −3.65)
RADS-2 somatic complaints	−2.13	−1.88	0.073	(−4.48, 0.21)	−5.14	−3.92	<0.001	(−7.88, −2.41)
MASC total	−2.41	−2.26	0.033	(−4.61, −0.21)	−5.37	−3.06	0.006	(−9.02, −1.73)
MASC physical symptoms	−0.52	−0.37	0.714	(−3.40, 2.36)	−3.81	−2.03	0.056	(−7.71, 0.10)
MASC harm/avoidance	−0.20	−0.12	0.902	(−3.58, 3.18)	1.05	0.44	0.667	(−3.96, 6.06)
MASC social anxiety	−3.76	−2.82	0.010	(−6.51, −1.00)	−5.04	−3.63	0.002	(−7.93, −2.15)
MASC separation/panic	−2.16	−2.03	0.054	(−4.35, 0.04)	−6.33	−3.13	0.005	(−10.52, −2.13)
ISI	−0.42	−0.44	0.665	(−2.40, 1.56)	−4.28	−3.60	0.002	(−6.77, −1.80)
CAMM	0.84	0.68	0.504	(−1.72, 3.40)	4.36	3.89	<0.001	(2.03, 6.68)
AFQ-Y	−6.50	−2.83	0.009	(−11.24, −1.75)	−7.00	−2.11	0.047	(−13.91, −0.09)
CDRS-R	−2.73	−1.81	0.082	(−5.84, 0.38)	−6.13	−3.51	0.002	(−9.78, −2.48)

*The measures reported are Reynolds Adolescent Depression Scale Second Edition (RADS-2), total *T* scores, and the subscales anhedonia/negative affect, dysphoric mood, negative self-evaluation, somatic complaints; Multidimensional Anxiety Scale for Children (MASC) total *T* scores, and the subscales physical symptoms, harm/avoidance, social anxiety, separation/panic; Insomnia Severity Index (ISI); Child and Adolescent Mindfulness Measure (CAMM); Avoidance and Fusion Questionnaire for Youth (AFQ-Y); interview assessment with Children’s Depression Rating Scale-Revised (CDRS-R)*.

### Secondary Outcomes

Interviewer assessed depression symptom severity by CDRS-R followed a similar but weaker pattern as the RADS-2. Between T0 and T1, a difference of mean score = −2.73, *t*-value = −1.81, *p* = 0.08, ES = 0.17, 95% CI −5.84 to 0.38; and from T0 to T2, a difference of mean score −6.13, *t*-value −3.51, *p* = 0.002, 95% CI: −9.78 to −2.48. Insomnia measured with ISI and mindfulness skills measured by the CAMM did not improve significantly during the intervention (T0−T1), but improved significantly when including the 3-month follow-up (T0–T2). ISI showed difference of mean scores T0–T2 of −4.28, *t*-value −3.6, *p* ≤ 0.002, 95% CI: −6.77 to −1.80. ISI was only used in Groups B and C. CAMM showed a difference of mean scores T0–T2 of 4.36, *t*-value = 3.89, *p* ≤ 0.001, 95% CI: 2.03 to 6.68. Psychological flexibility measured with AFQ-Y improved significantly during the TARA intervention and showed a change in mean scores from T0 to T1 of −6.50, *t* = −2.83, *p* = 0.0094, 95% CI and continued to improve slightly after the intervention was completed, delta = −7.00, *t* = −2.11, *p* = 0.047, 95% CI (Table 2; Figures 2C–F). ES for the *a priori* hypothesized specific proxy outcomes related to target 1–4, see Table 3.

**Table 3:** **Number of participants, means, SDs of assessments, and effect sizes (ES) calculated as proportion of variance pre-***Training for Awareness, Resilience, and Action*** (TARA) (T0), post-TARA (T1), and at 3 months after the end of TARA (T2)**.

	T0 *N* = 26	T1 *N* = 25	T2 *N* = 22	ES (proportion of variance)	ES (proportion of variance)
	
	Mean (SD)	Mean (SD)	Mean (SD)	T0–T1	T0–T2
RADS-2 total *T* scores	62.35 (10.59)	57.92 (9.50)	54.05 (9.94)	0.53	0.83
RADS-2 anhedonia/negative affect	58.96 (13.06)	54.52 (11.92)	52.18 (12.23)	0.27	0.27
RADS-2 dysphoric mood	60.50 (9.88)	58.08 (8.03)	54.27 (9.38)	0.09	0.39
RADS-2 negative self-evaluation	61.35 (11.65)	56.12 (9.02)	52.82 (8.97)	0.61	0.83
RADS-2 somatic complaints	59.62 (9.34)	57.44 (8.94)	54.27 (10.15)	0.15	0.73
MASC total *T* scores	64.73 (10.42)	62.28 (9.83)	59.77 (9.87)	0.21	0.45
MASC physical symptoms	58.07 (12.57)	57.36 (12.21)	54.68 (11.84)	0.01	0.20
MASC harm avoidance	54.12 (9.55)	53.80 (8.24)	55.05 (10.35)	0.00	0.01
MASC social anxiety	68.77 (7.45)	65.24 (10.84)	64.27 (9.81)	0.33	0.63
MASC separation panic	59.81 (11.22)	57.68 (10.35)	53.95 (9.53)	0.17	0.47
ISI	*N* = 21 10.82 (6.67)	*N* = 21 10.62 (6.82)	*N* = 20 6.68 (5.43)	0.01	0.67
CAMM	27.7 (7.53)	28.52 (9.24)	32.06 (7.23)	0.02	0.72
AFQ-Y	16.18 (7.17)	13.91 (7.68)	13.05 (7.68)	0.33	0.21
CDRS-R	66.62 (7.20)	63.92 (9.27)	60.65 (8.01)	0.17	0.38

*The measures reported are Reynolds Adolescent Depression Scale Second Edition (RADS-2) total *T* scores, Multidimensional Anxiety Scale for Children (MASC) total *T* scores, Insomnia Severity Index (ISI), Child and Adolescent Mindfulness Measure (CAMM), Avoidance and Fusion Questionnaire for Youth (AFQ-Y), and interview assessment with Children’s Depression Rating Scale-Revised (CDRS-R)*.

### Medication Changes

No changes of medications were made as part of the TARA protocol. No participants added additional antidepressant or stimulant medications during TARA. Discontinuations of medication during TARA were reported, and whether this was related to TARA is uncertain. Three of the 17 participants who were taking antidepressant medication at T0 stopped taking this medication by T2 and one had reduced the number of antidepressant medications from 2 to 1. Two participants had stopped taking stimulant medication at T2. Some additional information with regard to the discontinuation of medication was obtained at the T2 interview. One participant had lost her medication during the summer holiday and in the T2 interview she said it was likely she would begin taking the medication again, but it is unknown whether she did. Another participant never took her medication during summer holiday and planned to begin again when school started.

### Monitoring Assessment

The Children’s Outcome Rating Scale (CORS) indicated an overall improvement in well-being over the course of the intervention, with participant mean score increasing from 21.1 to 30.6 (out of maximum 40) from session 1 to 12. This measure was intended as a monitoring instrument to identify unexpected decreases in well-being during the intervention and not as an outcome measure and therefore was not further analyzed. The Children’s Session Rating Scale (CSRS), which was administered after every session, reflected overall satisfaction with the content with a mean total score of 33.5 out of maximum 40. During sessions 1–12, the CSRS scores ranged from 25.9 to 37.3.

### Qualitative Data from Focus Group Interviews

All participants were invited to the focus group interview with a trained interviewer that took place shortly after the last session. Many participants reported that they had chosen to participate in TARA because it was different from the “talk-therapy,” which they already had experienced. Participants reported that they liked the diversity of activities and skills taught in the classes, with some who specifically enjoyed the yoga-based movement. Some of the participants valued the opportunity to meet teens in similar situations as themselves. In general, they perceived the group as a safe place where they felt listened to and respected. They reported liking the format, duration, and frequency of the sessions and wanted more interactive practices and discussions. Most participants said that they especially found the breathing exercises helpful, that they practiced these outside of class and reported benefits in terms of anxiety- and stress reduction, as well as improved sleep. Participants reported that they gradually became more appreciative of doing practices in silence over the 12 weeks. Some of the participants were less enthusiastic about the presentation of the scientific rationale, while others found this educational component specifically helpful.

## Discussion

This single-arm pilot study was conducted to test the feasibility, acceptability, and preliminary efficacy of a novel treatment for adolescent depression, *TARA*. We found that TARA was feasible and acceptable in terms of study retention (84.6%), attendance rates (in average 9.5/12 sessions), and from positive post-TARA focus group feedback regarding the structure and content of the program. The results demonstrated preliminary efficacy of the TARA program in improving symptoms of depression and generalized and social anxiety. Importantly, these symptoms continued to improve after the program, suggesting successful acquisition and retention of adequate emotion regulation skills and a willingness to use the practices in daily life after the TARA program ended.

The specific aim of this study was to test the scientific hypotheses regarding the targeted neural mechanisms of change in the four modules of TARA, using specific symptom and behavioral indicators as clinically relevant proxies. We assessed the change in outcome measures from baseline (T0) to post-intervention (T1) and from baseline to 6-month follow-up (T2).

The strategy to impact *the first target* of amygdala hyperreactivity was to promote vagal and sensory afference through breathing practices and slow synchronized movement. We hypothesized that this would correspond to a decrease of somatic and vegetative symptoms of depression and improved self-regulatory skills and sleep. These symptoms are also common in generalized forms of anxiety including stomach aches, feeling ill, fatigue, insomnia, or other sleep disturbances. These symptoms are well defined in the somatic complaints subscale of RADS-2. We found preliminary evidence that this first target of sustained amygdala hyperreactivity was influenced through the intervention, based on the decrease the scores of RADS-2 Somatic Complaints subscale, as well as on the improvement of self-regulatory skill measured by CAMM, and reduced insomnia measured with ISI (Table 2). It may be noted that the MASC subscale Physical Symptoms captures symptoms that are more common in acute forms of anxiety such as panic attacks or specific phobias, including feeling tense or restless, having trouble breathing, feeling dizzy, having sweaty or cold hands, being shaky or jittery. These symptoms belong to the RDoC construct of acute threat and were not targeted in the TARA program. Similarly, the symptoms assessed in the MASC subscales of panic or harm/avoidance were not specifically targeted in TARA and did not show significant change pre- compared to post-intervention. These findings support the specificity of the TARA intervention in reducing symptoms relevant to the first target—the sustained limbic hyperreactivity.

Our strategy to impact *the second target*, inflexibility in the ability to shift neural activity away from DMN, was to practice noticing negative self-referencing processing and then consciously shifting to present moment sensory and interoceptive awareness. We hypothesized that this would decrease rumination as measured by RADS-2 Negative Self-Evaluation, which was supported in the study.

*The third target*, interpersonal stress and social anxiety, was addressed by bringing the acquired skillset of emotion regulation from modules 1 to 2 into the context of social interaction. Participants practiced labeling and expressing emotions, identifying emotional triggers, empathetic listening, and a compassionate response to oneself and others. We hypothesized that these practices would result in a decrease of social anxiety, which was supported by significant changes in the MASC subscale Social Anxiety. It should be pointed out that social anxiety has not been considered as a potential confounding factor for changes in depression symptom severity pre- compared to post-TARA, but rather as an expression of a dysfunction in the same RDoC construct, here being expressed within the same unit of analysis.

*The fourth target* was to strengthen cognitive control by introducing behavioral activation guided by intrinsic reward. During the fourth module, time was allocated to defining a life purpose, and discussions and practices were aimed at both learning how to stay on a trajectory toward this purpose and challenging patterns of experiential avoidance by top-down cognitive control over affective responses. The preliminary efficacy of module four was less evident compared to previous modules. The change in scores pre- to post TARA of the Anhedonia Subscale of RADS-2 and of AFQ-Y, which measures experiential avoidance, committed action and behavioral effectiveness in the presence of distressing emotional experiences, were statistically significant, but showed smaller effect sizes. We speculate that this may be explained by the reduced time allocated for these practices, because they were introduced toward the end of the curriculum, in session 10.

In summary, this detailed breakdown of symptoms and behavioral outcomes allowed us to investigate the question of whether we were effectively targeting mechanisms relevant to clinical improvement of adolescent depressive symptoms. Our results provide initial support for three of the targets, with large effects observed for physical symptoms of depression, insomnia, negative self-referential processing, and social anxiety. The last target of cognitive control may have had insufficient time allocated in the curriculum.

The qualitative data obtained from the focus group suggest that the participants perceive that they were helped by the self-regulatory practices and that they continued to practice after the TARA program ended. This may help prevent future recurrent episodes of depression. The CSRS showed that participants generally thought the content of the sessions was relevant and important. The gradual improvement of emotional well-being measured at each session with the CORS also supports the progression of skills uptake over the 12 weeks of active TARA intervention.

It has been suggested that amygdala hyperreactivity could be a mediator between adverse childhood experiences and the development of emotional disorders (66). We conducted baseline assessment of early trauma (CTQ) and verified a positive correlation between CTQ and depression symptoms (RADS-2) at baseline (*r* = 0.52, *p* < 0.01), which has been consistently found in previous studies (33, 67). However, CTQ at baseline was not correlated with reduction of RADS-2 scores from T0 to T2, suggesting that childhood trauma did not moderate the effect of the TARA treatment in reducing depression symptoms.

Our pilot study had several limitations. First, the sample size was small and we had no control condition. The TARA intervention was developed on the basis of fMRI findings of adolescent depression within the framework of the NIMH RDoC criteria and this pilot study could not directly test our hypothesis of brain change. The size of the Groups A–C varied and the teachers differed in Groups A and B versus C. The study design did not allow us to fully assess whether this had an impact on the TARA outcomes. We did not fully assess the frequency and duration of the home practice, which would have been interesting to relate to the outcome.

A proof-of-concept MRI study to more directly test our hypothesis of brain changes is ongoing and if the hypotheses are shown valid a next step would be to conduct a well-powered randomized controlled trial, preferably with an active control condition. We are continuously collecting additional qualitative data from new focus groups, to allow more in depth analyses and a deeper qualitative assessment of our work. Since the group format of focus group interviews may allow for a possible influence of opinions, within the group of participants, we are also, in future TARA groups, adding qualitative assessment by an observer blinded to the nature of the intervention attending the full scope of classes. In these future groups, we also intend adding separate post-TARA interviews of the parents/legal guardians of the participants. We hope this may lead to further refinement of the TARA manual, including the allocation of more time to module four targeting cognitive control, as well as parental education groups to support the participants between sessions. Additionally, we are currently working on a systematic teacher training protocol to manage larger studies.

In conclusion, we demonstrated that the neuroscientifically based TARA program was both feasible and acceptable in a sample of clinically depressed and/or anxious adolescents and could be delivered with fidelity. We established preliminary efficacy as indicated by improvement of depression symptom and social anxiety scores pre- to post-TARA, importantly also in cases that had not responded to SSRIs and/or psychological treatment for more than 6 months prior to entering the TARA program. Furthermore, the participants showed maintained reduction in depressive symptoms and social anxiety 3 months after TARA ended, suggesting maintained skills after the program had ended. We also showed that the specific symptom and behavioral outcomes corresponded well with the hypothesized mechanisms of change.

We acknowledge the complexities of RDoC-based translational research and hope this article may be useful as a template for helping others apply RDoC-based theories in the development of clinical applications and thereby provide patients suffering from psychiatric disorders with targeted and more effective treatments.

## Author Contributions

Designed and developed the TARA program: EH. Designed the study: EH, LD, FH, PM, TY, TH, MC, and HW. Performed data acquisition: EH, VG, PM, and LB. Ethical considerations: EH, PM, LD, TY, and FH. Performed data cleaning and analyses: EH, MC, VG, and MA. Interpreted and framed the qualitative data: EH, MC, TY, and LB. Interpreted the data: EH, OT, TH, MA, MC, FH, and TY. Literature review: EH, OT, TH, and CC. Wrote the paper: EH, OT, TH, CC, MC, FH, and TY.

## Conflict of Interest Statement

The authors declare that the research was conducted in the absence of any commercial or financial relationships that could be construed as a potential conflict of interest.
