# Supplementary material for: Feasibility and Preliminary Efficacy of a Novel RDoC-Based Treatment Program for Adolescent Depression: “Training for Awareness Resilience and Action” (TARA)—A Pilot Study
Source: Front Psychiatry. 2017 Jan 16;7:208. doi: 10.3389/fpsyt.2016.00208 (PMC5237634; doi:10.3389/fpsyt.2016.00208)
Supplement: Supplementary file 1 [file Image_1.PDF]

Supplementary material for:

Feasibility and preliminary efficacy of a novel RDoC-based treatment program for adolescent depression: Training for Awareness Resilience and Action (TARA) – a pilot study

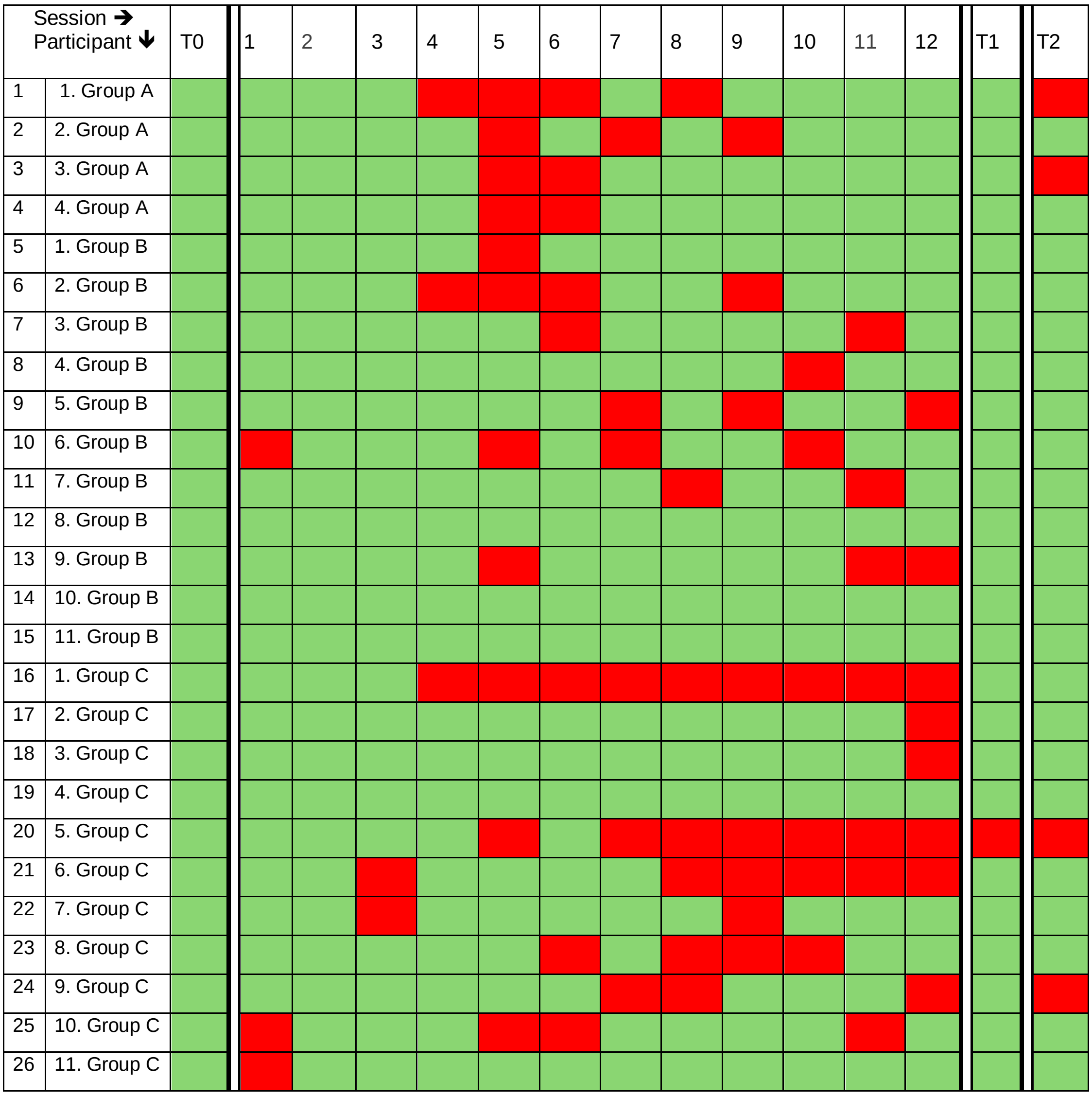

Figure S1. Diagram showing *Training for Awareness, Resilience and Action* (TARA) session attendance and assessment participation, green=present, red=absent.

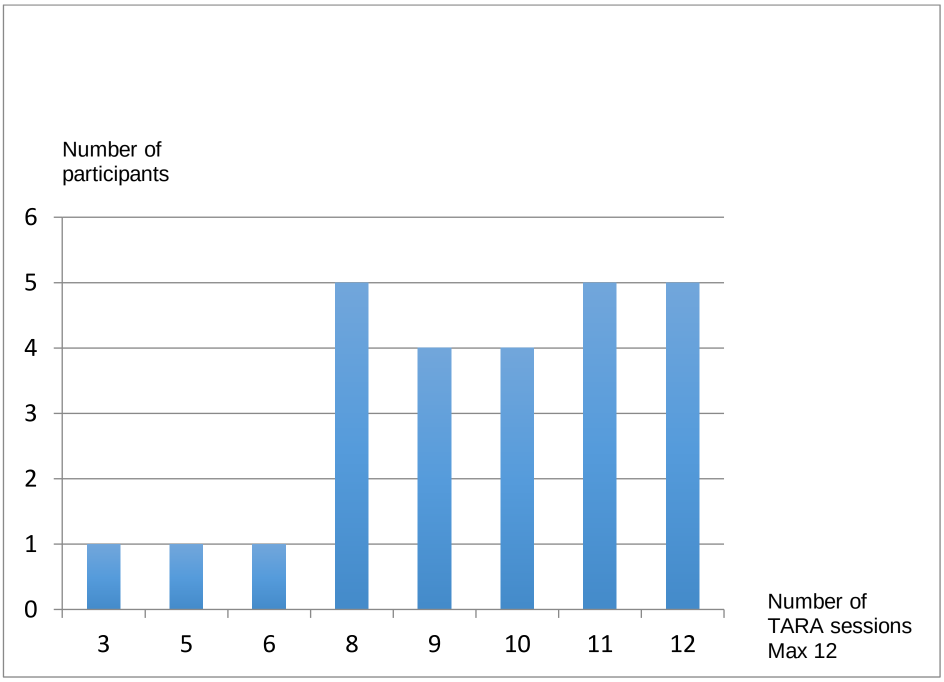

Figure S2. Number of sessions of *Training for Awareness, Resilience and Action* (TARA) attended by the participants.

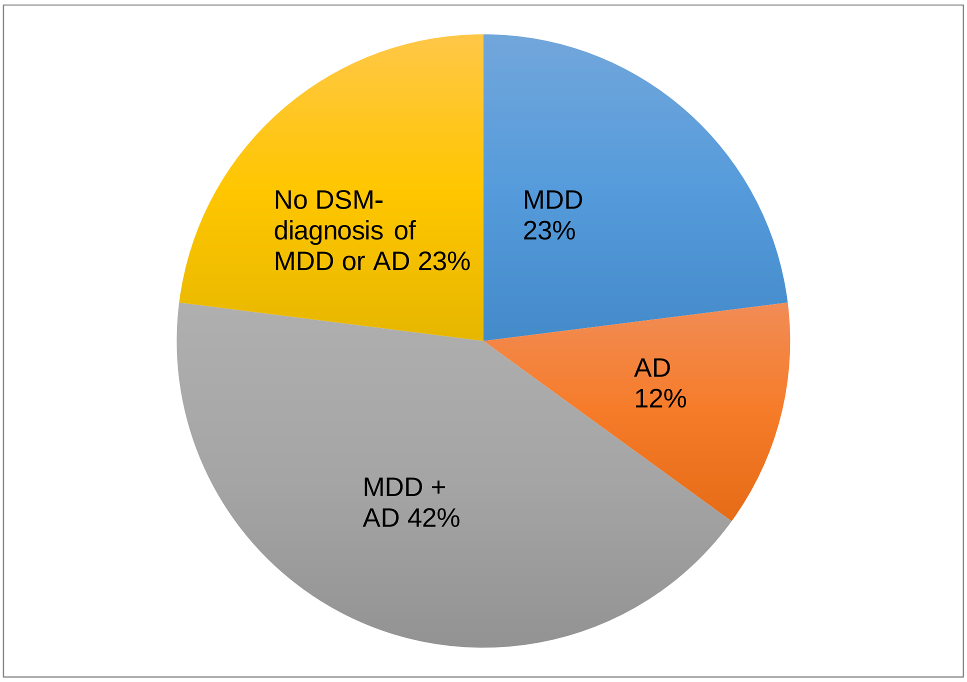

Figure S3. Distribution of the preliminary DSM-IV diagnoses at baseline, as assessed by the Development and Well-Being Assessment of Major Depressive Disorder (MDD) and Anxiety Disorders (AD) including generalized anxiety disorder, social anxiety disorder, panic disorder, specific phobias.
